# Supplementary material for: Time-lapse crystallography snapshots of a double-strand break repair polymerase in action
Source: Nat Commun. 2017 Aug 15;8:253. doi: 10.1038/s41467-017-00271-7 (PMC5557891; doi:10.1038/s41467-017-00271-7)
Supplement: Supplementary file 1 — Supplementary Information [file 41467_2017_271_MOESM1_ESM.pdf]

File name: Supplementary Information
Description: Supplementary figures and supplementary table.

File name: Peer review file
Description:

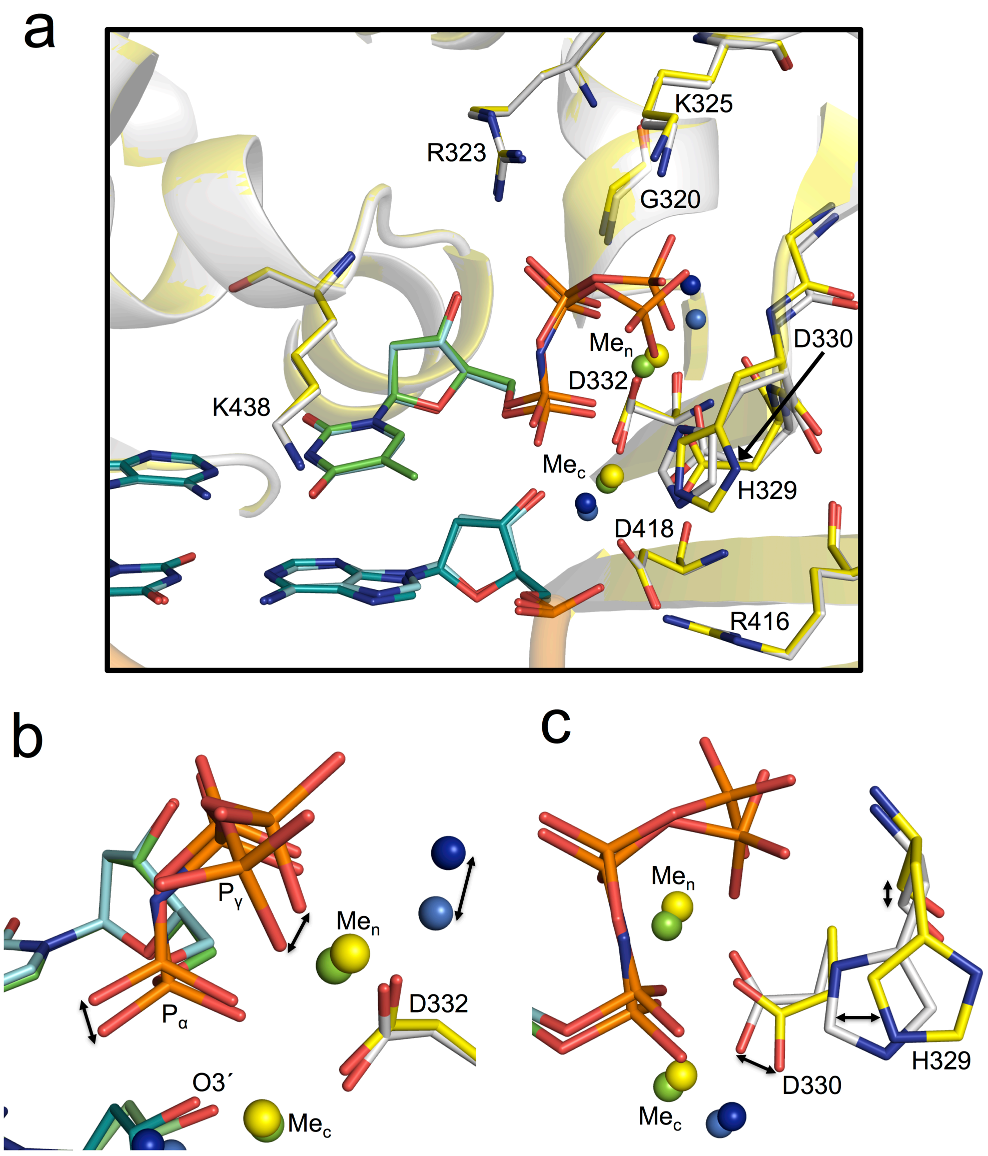

Supplementary Figure 1. Comparison of pol μ pre-catalytic ternary complexes. (**a**) The active site of the pol μ ground-state (GS) ternary complex bound to TTP (green carbons) and Ca^{2+} (yellow spheres) overlayed with a ternary complex bound to a non-hydrolyzable nucleotide (dUMPNPP, cyan carbons) and Mg^{2+} (green spheres; PDB ID 4M04). Side-chains are colored in yellow and light grey for the Ca^{2+}- and dUMPNPP-bound complexes, respectively. Me_c and Me_n refer to the catalytic and nucleotide metals, respectively. (**b**) Focused view highlighting differences in the position of the triphosphate of the incoming nucleotide. The water associated with Ca_n is shown as a dark blue sphere, while the Mg_n associated water is in light blue. The primer terminus is indicated (O3´). (**c**) Changes in His329 (H329) and Asp330 (D330). Black arrows highlight differences between the structures.

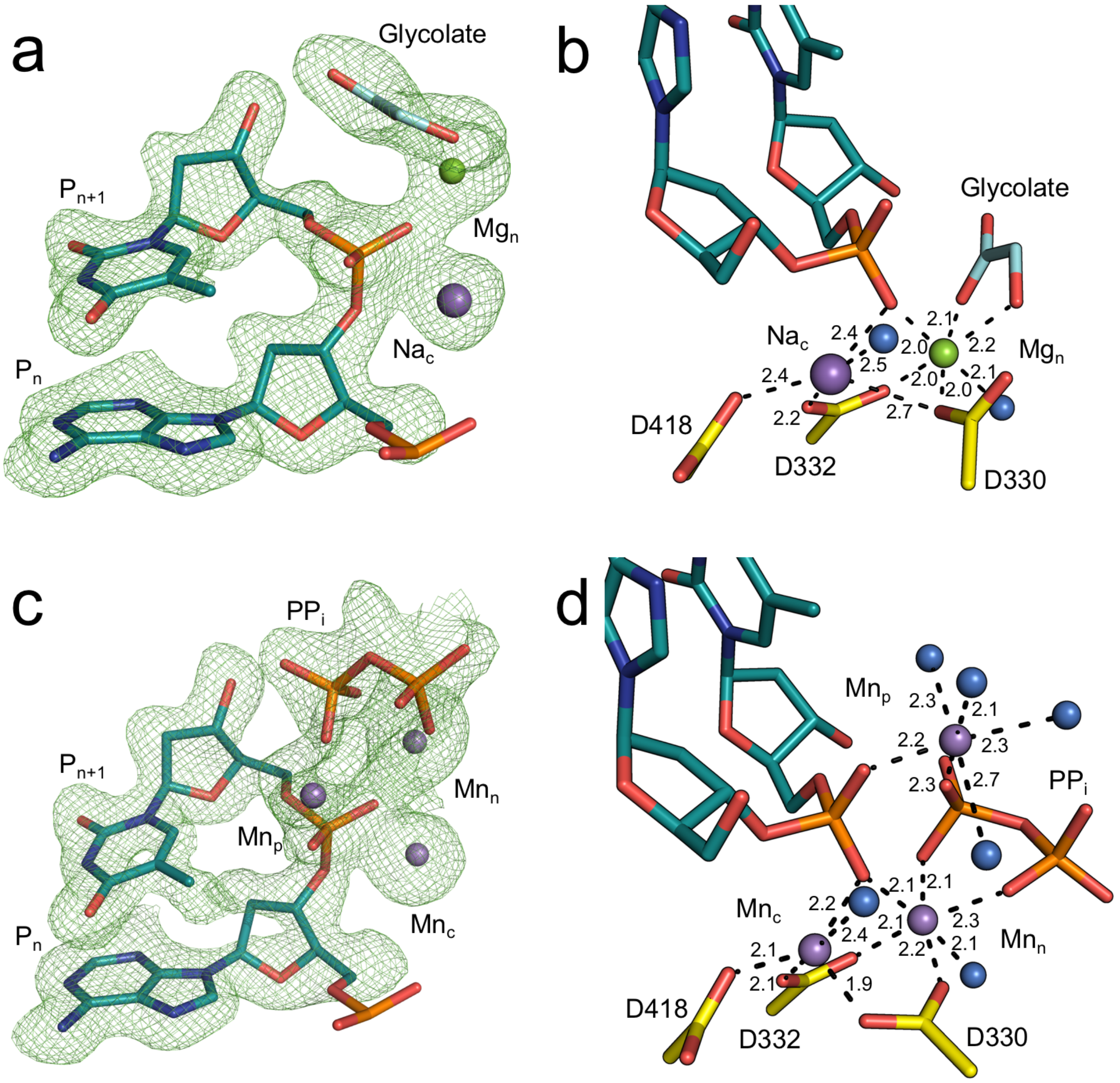

Supplementary Figure 2. Product complexes after varying soak times in $MgCl_2$ or $MnCl_2$. (**a,b**) Product complex after the 270 min soak in the presence of 10 mM $MgCl_2$. (**c,d**) Product complex after the 60 min soak with 10 mM $MnCl_2$. Panels (**a**) and (**c**) display F_o-F_c simulated annealing omit density (green mesh) contoured at 2.5σ for the primer terminus (P_n), incorporated (P_{n+1}) nucleotide and active site metal ions. Mg_n and $Mn_{(c,n,p)}$ are displayed as green and magenta spheres, respectively. Na_c is a larger purple sphere. The view in panels (**b**) and (**d**) is a 90° rotation from the view on the left. Metal coordination is shown with dashed lines and distances (Å) are indicated. Blue spheres are metal-coordinating water. The active site aspartates (D330, D332, D418) are in yellow stick representation, while DNA is in cyan. Glycolate (light blue) was modeled into the density vacated by PP_i in the 270 min Mg soak.

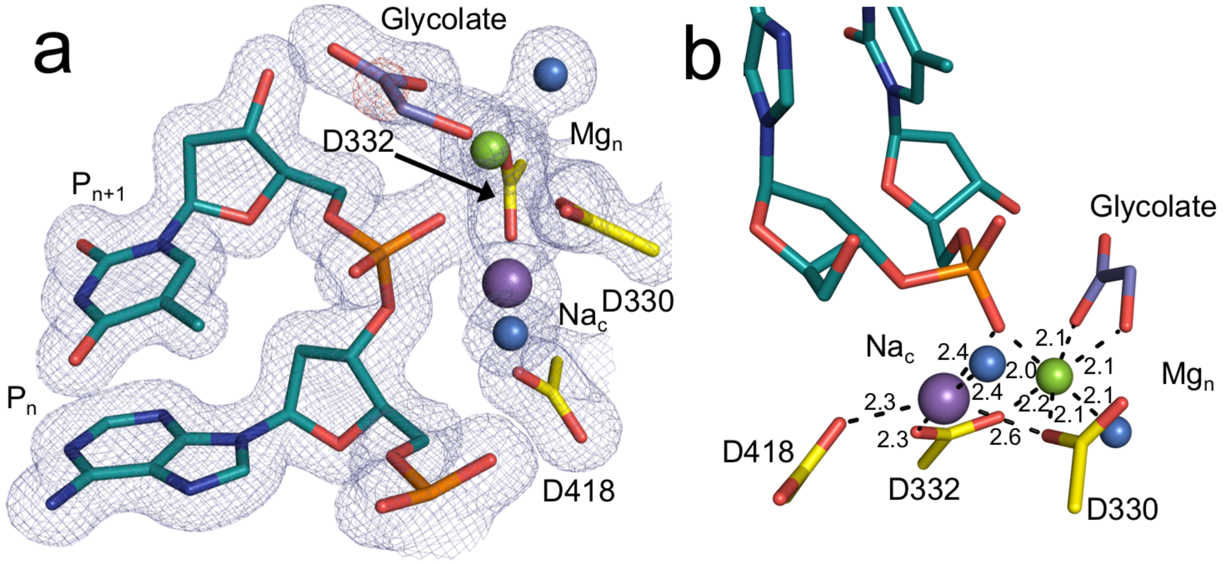

Supplementary Figure 3. Product complex after a 960 min magnesium soak. The pol μ ground state (GS) ternary complex crystal was soaked in a cryo-solution containing 10 mM $MgCl_2$ for 960 min. (**a**) The $2F_o$-F_c map (blue mesh) shown is contoured at 2σ and overlayed with an F_o-F_c map contoured at 3σ. The latter is displayed in green for positive and red for negative difference density. Glycolate (purple stick) was modeled into density near the nucleotide metal. (**b**) A 90° rotation of (**a**) showing metal coordination (dashed lines) and distances (Å). The newly formed (P_{n+1}) and previous primer terminus (P_n) are shown in stick representation (cyan), while the protein side chains are yellow. Mg^{2+} ions and water molecules are displayed as green and blue spheres, respectively. Sodium is shown as a purple sphere.

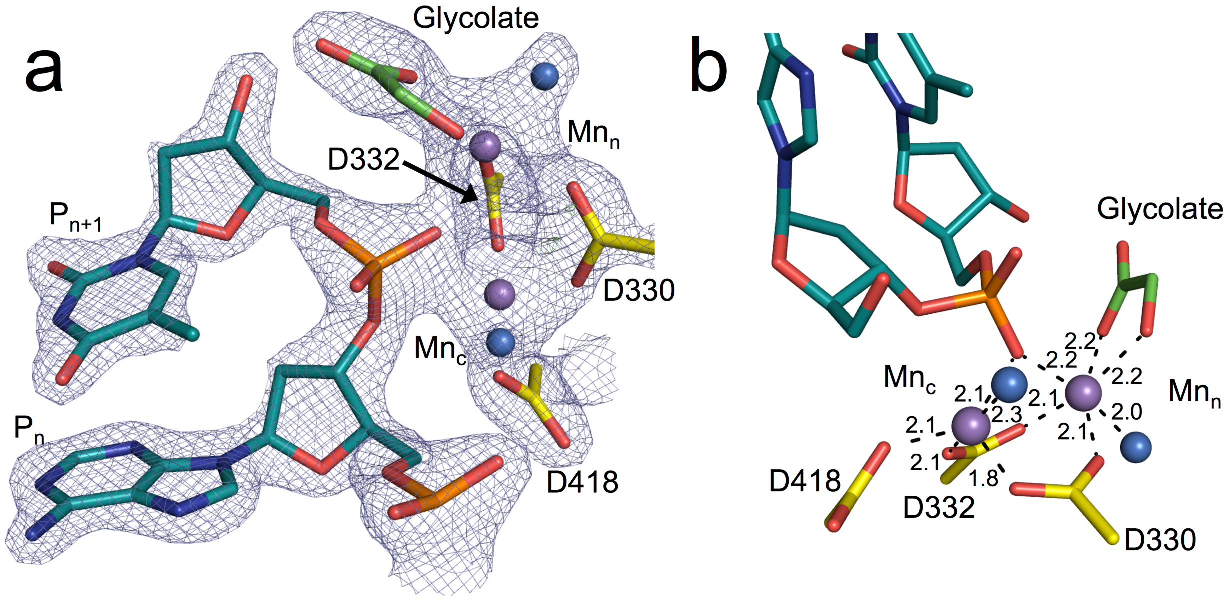

Supplementary Figure 4. Product complex after a 960 min manganese soak. The pol µ ground state (GS) ternary complex crystal was soaked in a cryo-solution containing 10 mM $MnCl_2$ for 960 min. (**a**) The $2F_o$-F_c map (blue mesh) shown is contoured at 2σ and overlayed with an F_o-F_c map contoured at 3σ. The latter is displayed in green (positive) and red (negative) difference density. Glycolate (green stick) was modeled into density near the nucleotide metal. (**b**) A 90° rotation of (**a**) showing metal coordination (dashed lines) and distances (Å). The incorporated nucleotide (P_{n+1}) and previous primer terminus (P_n) are in cyan stick representation, while the protein side-chains are yellow. Mn^{2+} ions and water molecules are displayed as purple and blue spheres, respectively.

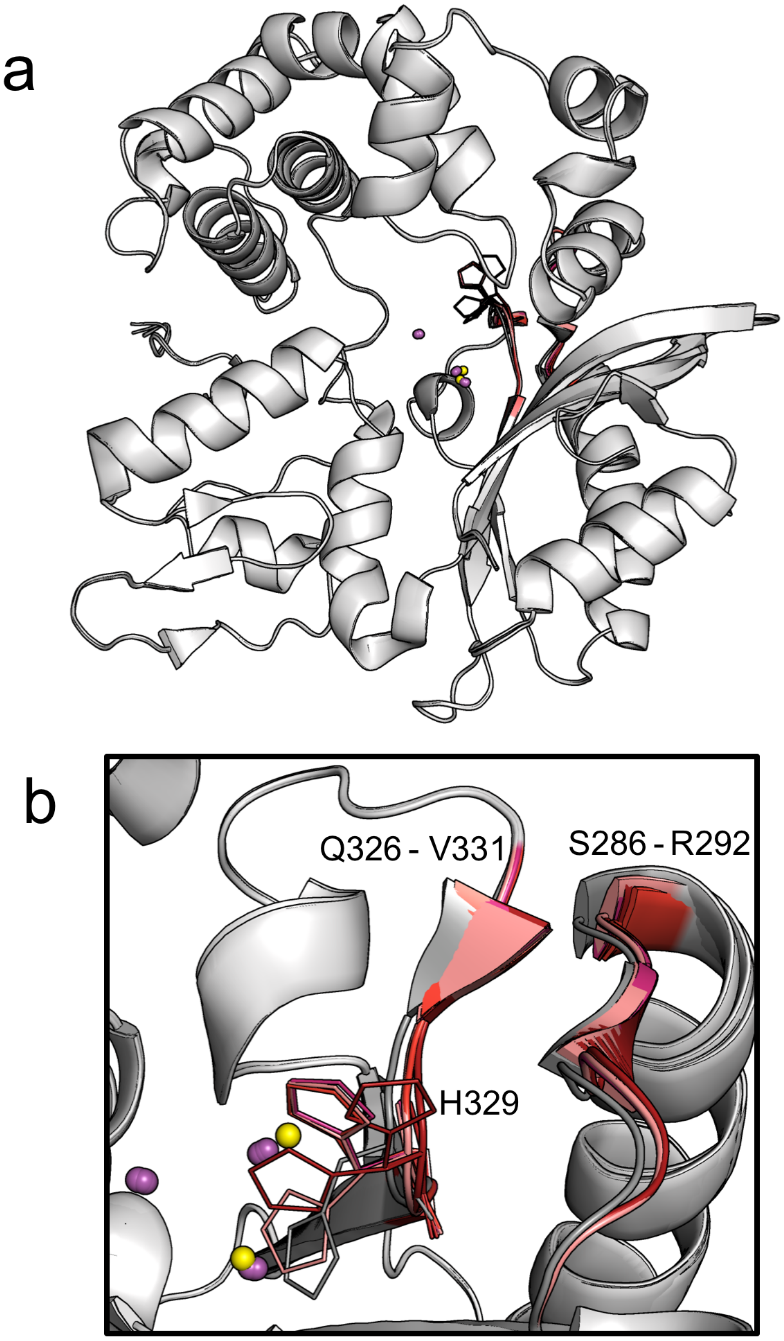

Supplementary Figure 5. Global conformational changes in pol μ during Mn^{2+}-mediated catalysis. (**a**) Superimposed ribbon representations of the ground state (GS) Ca^{2+} structure with the Mn^{2+} (RS), (PS) and (PS*) structures. Sections of the protein backbone that display movement, as the reaction progresses (GS—PS*), are colored in increasingly darker shades of red. Changes in His329 (H329) are shown in wire representation. The metals in the catalytic site are displayed as yellow and magenta spheres for Ca^{2+} and Mn^{2+}, respectively. (**b**) A focused view around His329 highlighting shifts (~1 Å) in neighboring loops (Gln326-Val331 and Ser286-Arg292). The superimpositions were performed using PyMol.

Supplementary Table 1. Crystallographic and refinement statistics.

	10 mM Ca, 15 min	10 mM Mg, 7.5 min	10 mM Mg, 15 min	10 mM Mg, 45 min	10 mM Mg, 60 min
**PDB ID**	5TXX	5TYB	5TYC	5TYD	5TYE
**Data Collection**					
Space Group	$P2_12_12_1$	$P2_12_12_1$	$P2_12_12_1$	$P2_12_12_1$	$P2_12_12_1$
Cell Dimensions					
a, b, c (Å)	59.538	60.047	60.004	59.891	60.000
	68.243	68.488	68.577	68.590	68.573
	110.200	110.450	110.373	110.476	110.483
α, β, γ (°)	90, 90, 90	90, 90, 90	90, 90, 90	90, 90, 90	90, 90, 90
Resolution (Å)	50 - 1.95	50 - 1.85	50 - 2.10	50 - 1.90	50 - 2.02
R_{sym} or R_{merge} [1]	7.9 (42.7)	8.3 (57.4)	11.9 (50.9)	7.0 (96.3)	9.8 (57.7)
$I/\sigma I$ [1]	16.2 (2.2)	20.4 (1.9)	15.5 (2.9)	30.0 (2.2)	12.2 (2.0)
Completeness (%)[1]	99.0 (90.2)	99.6 (95.0)	99.8 (99.4)	100.0 (100.0)	98.8 (98.9)
Redundancy[1]	4.7 (3.8)	5.9 (4.0)	6.6 (4.8)	7.4 (7.4)	3.4 (3.1)
No. Unique Reflections[1]	33289	39693	29688	36710	30701
**Refinement**					
RS : PS (%)	100 : 0	70 : 30	50 : 50	40 : 60	0 : 100
A site occupancy (%)	100 Ca^{2+}	100 Ca^{2+}	50 Ca^{2+} 50 Mg^{2+}	100 Mg^{2+}	30 Mg^{2+} 70 Na^{+}
B site occupancy (%)	100 Ca^{2+}	100 Mg^{2+}	100 Mg^{2+}	100 Mg^{2+}	100 Mg^{2+}
C site occupancy (%)	–	–	–	–	–
Resolution (Å)	1.95	1.85	2.10	1.90	2.05
No. reflections	32898	48659	27022	36947	28864
R_{work}/R_{free}	0.16 / 0.20	0.17 / 0.20	0.20 / 0.24	0.17 / 0.21	0.19 / 0.23
No. atoms					
Protein / DNA	2709 / 385	2745 / 426	2680 / 426	2706 / 426	2670 / 405
TTP / PP_i / Metal	29 / 0 / 3	29 / 9 / 3	29 / 9 / 4	29 / 9 / 3	0 / 9 / 4
Water / Solutes	331 / 18	393 / 25	323 / 10	354 / 21	319 / 22
B-factors					
Protein and DNA	22.5	22.5	26.2	21.8	26.9
Me_A / Lig_A	13.2 / 16.3	18.2 / 18.3	20.7 / 22.6	12.2 / 15.9	17.3 / 19.9
Me_B / Lig_B	12.8 / 14.3	12.9 / 16.3	17.9 / 22.6	12.5 / 16.1	19.4 / 21.8
Me_C / Lig_C	–	–	–	–	–
Water / Solutes	31.6 / 25.1	30.1 / 25.9	31.1 / 25.9	28.5 / 25.5	30.6 / 33.9
Wilson B	21.5	20.2	24.9	19.1	25.3
R.M.S Deviations					
Bond Lengths (Å)	0.007	0.007	0.003	0.007	0.003
Bond Angles (°)	0.947	0.937	0.633	0.898	0.517

[1]Data in the highest resolution shell is in parentheses.

	10 mM Mg, 270 min	10 mM Mg, 960 min	100 mM Mg, 15 min	10 mM Mn, 4 min
**PDB ID**	5TYF	5TYG	5TXZ	5TYU
**Data Collection**				
Space Group	$P2_12_12_1$	$P2_12_12_1$	$P2_12_12_1$	$P2_12_12_1$
Cell Dimensions				
a, b, c (Å)	59.970	59.998	59.901	60.285
	68.468	68.534	68.572	62.318
	110.654	110.747	110.498	118.546
α, β, γ (°)	90, 90, 90	90, 90, 90	90, 90, 90	90, 90, 90
Resolution (Å)	50 - 1.97	50 - 1.73	50 - 1.65	50 - 2.05
R_{sym} or R_{merge}[1]	10.4 (75.3)	5.5 (90.9)	6.3 (71.2)	9.4 (85.7)
$I/\sigma I$[1]	17.1 (2.0)	36.8 (2.1)	24.9 (2.1)	19.3 (2.1)
Completeness (%)[1]	99.8 (97.8)	100.0 (100.0)	100.0 (100.0)	99.0 (98.3)
Redundancy[1]	6.7 (4.7)	7.3 (7.3)	4.3 (4.3)	5.5 (5.5)
No. Unique Reflections[1]	32965	48737	55443	28899
**Refinement**				
RS : PS (%)	0 : 100	0 : 100	50 : 50	60 : 40
A site occupancy (%)	100 Na^+	100 Na^+	40 Ca^{2+}<br>60 Mg^{2+}	60 Ca^{2+}<br>40 Mn^{2+}
B site occupancy (%)	100 Mg^{2+}	100 Mg^{2+}	100 Mg^{2+}	100 Mn^{2+}
C site occupancy (%)	–	–	–	40 Mn^{2+}
Resolution (Å)	1.97	1.73	1.65	2.05
No. reflections	32627	48659	55352	28577
R_{work}/R_{free}	0.17 / 0.21	0.16 / 0.19	0.17 / 0.19	0.19 / 0.21
No. atoms				
Protein / DNA	2725 / 405	2745 / 405	2792 / 426	2676 / 385
TTP / PP_i / Metal	0 / 0 / 3	0 / 0 / 3	29 / 9 / 4	29 / 9 / 6
Water / Solutes	355 / 19	385 / 22	444 / 33	248 / 18
B-factors				
Protein and DNA	30.1	31.5	32.8	31.8
Me_A / Lig_A	20.7 / 19.0	23.4 / 22.4	12.8 / 18.3	25.9 / 28.9
Me_B / Lig_B	17.4 / 19.7	18.0 / 21.2	14.6 / 15.5	28.1 / 26.4
Me_C / Lig_C	–	–	–	36.3 / 28.4
Water / Solutes	37.4 / 36.9	39.8 / 40.2	32.9 / 26.9	36.7 / 34.5
Wilson B	26.6	24.6	19.4	30.7
R.M.S Deviations				
Bond Lengths (Å)	0.005	0.007	0.007	0.003
Bond Angles (°)	0.701	0.876	0.960	0.571

	10 mM Mn, 7.5 min	10 mM Mn, 10 min	10 mM Mn, 15 min	10 mM Mn, 60 min	10 mM Mn, 960 min
**PDB ID**	5TYV	5TYW	5TYX	5TYY	5TYZ
**Data Collection**					
Space Group	$P2_12_12_1$	$P2_12_12_1$	$P2_12_12_1$	$P2_12_12_1$	$P2_12_12_1$
Cell Dimensions					
a, b, c (Å)	59.977	59.825	59.996	60.031	59.940
	68.791	68.521	68.785	68.709	68.669
	109.998	109.825	110.093	110.211	110.006
α, β, γ (°)	90, 90, 90	90, 90, 90	90, 90, 90	90, 90, 90	90, 90, 90
Resolution (Å)	50 - 1.93	50 - 1.88	50 - 1.95	50 - 1.95	50 - 1.98
R_{sym} or R_{merge}[1]	7.2 (73.9)	5.9 (61.5)	8.7 (63.7)	7.0 (62.5)	7.7 (59.6)
$I/\sigma I$[1]	31.7 (1.7)	24.4 (1.8)	28.5 (1.9)	33.3 (2.4)	28.0 (1.9)
Completeness (%)[1]	99.4 (93.2)	99.8 (98.4)	99.1 (92.1)	99.5 (95.1)	99.4 (94.3)
Redundancy[1]	9.7 (5.3)	6.0 (3.6)	10.4 (5.1)	10.3 (6.4)	7.8 (4.4)
No. Unique Reflections[1]	34940	37780	33894	30701	32266
**Refinement**					
RS : PS (%)	40 : 60	30 : 70	0 : 100	0 : 100	0 : 100
A site occupancy (%)	100 Mn^{2+}	100 Mn^{2+}	100 Mn^{2+}	100 Mn^{2+}	100 Mn^{2+}
B site occupancy (%)	100 Mn^{2+}	100 Mn^{2+}	100 Mn^{2+}	100 Mn^{2+}	100 Mn^{2+}
C site occupancy (%)	50 Mn^{2+}	60 Mn^{2+}	60 Mn^{2+}	40 Mn^{2+}	–
Resolution (Å)	1.93	1.88	1.95	1.95	1.98
No. reflections	34504	37655	32642	33918	31225
R_{work}/R_{free}	0.19 / 0.22	0.18 / 0.21	0.19 / 0.22	0.19 / 0.22	0.18 / 0.22
No. atoms					
Protein / DNA	2687 / 426	2713 / 426	2661 / 405	2686 / 405	2697 / 405
TTP / PP_i / Metal	29 / 9 / 8	29 / 9 / 7	0 / 9 / 8	0 / 9 / 8	0 / 0 / 7
Water / Solutes	340 / 17	377 / 22	350 / 15	367 / 10	312 / 24
B-factors					
Protein and DNA	28.9	28.2	30.0	28.1	35.6
Me_A / Lig_A	23.3 / 23.6	23.0 / 24.8	24.0 / 23.3	21.6 / 20.5	28.8 / 28.7
Me_B / Lig_B	21.7 / 21.0	24.3 / 24.2	19.9 / 21.5	17.4 / 20.4	23.3 / 26.1
Me_C / Lig_C	35.2 / 23.1	44.3 / 29.2	43.4 / 27.3	33.3 / 24.7	–
Water / Solutes	33.9 / 29.2	35.4 / 35.5	36.4 / 34.8	32.5 / 35.2	46.4 / 42.7
Wilson B	18.4	25.0	18.7	19.5	23.8
R.M.S Deviations					
Bond Lengths (Å)	0.003	0.006	0.005	0.008	0.005
Bond Angles (°)	0.541	0.752	0.729	0.958	0.680
